# Insights Into Abdominal Wall Vascularized Composite Allotransplantation: An Individual Patient Data Analysis

**DOI:** 10.1002/micr.70346

**Published:** 2026-11-14

**Authors:** Mohammadsadegh Sabagh, Nastaran Sabetkish, Mohammadamin Shahrbaf, Elias Khajeh, Christoph Michalski, Arianeb Mehrabi, Ulrich Kneser, Christoph Koepple

**Affiliations:** ^1^ Department of General, Visceral and Transplantation Surgery Heidelberg University Hospital Heidelberg Germany; ^2^ Department of Plastic, Hand and Reconstructive Surgery BG Trauma Center Ludwigshafen Ludwigshafen Germany; ^3^ Department of Hand and Plastic Surgery University of Heidelberg Heidelberg Germany

**Keywords:** Abdominal wall vascularized composite allotransplantation, intestinal transplantation, reconstructive surgery

## Abstract

**Background:**

Abdominal wall vascularized composite allotransplantation (AWT) has emerged as a reconstructive option for patients with complex abdominal wall defects, particularly in conjunction with intestinal transplantation. Despite more than 2 decades of clinical experience, the available evidence remains limited to isolated case reports and small case series, and important questions regarding patient selection, operative techniques, immunosuppressive strategies, rejection patterns, and clinical outcomes remain unanswered. Therefore, we performed an individual patient data analysis to comprehensively evaluate the current evidence and provide insights into the clinical applicability, efficacy, and limitations of AWT.

**Methods:**

We conducted a systematic review with an individual patient data analysis in accordance with the Preferred Reporting Items for Systematic Reviews and Meta‐Analyses (PRISMA) statement. PubMed, Web of Science, Scopus, and Embase were searched through December 2025.

**Results:**

Twenty studies published between 2003 and 2023 reported 35 individual patients who underwent 37 AWT procedures. The median patient age was 37.5 years (IQR, 17.9), and follow‐up ranged from 1 to 7 years. Alemtuzumab was used for induction in 32 patients (91.4%). The principal indications were large abdominal wall defects in 17 patients (48.6%), malignancy in 11 (31.4%), motility disorders in three (8.6%), and vascular disorders and congenital defects in two patients each (5.7%). All AWT procedures were performed in conjunction with intestinal transplantation; 23 patients (65.7%) received isolated intestinal transplantation and 12 (34.3%) received multivisceral transplantation. Thirteen patients (37.2%) developed biopsy‐proven AWT rejection. None of the assessed factors was significantly associated with rejection (all *p* > 0.05).

**Conclusion:**

AWT is a feasible reconstructive option for selected patients with extensive abdominal wall defects undergoing intestinal or multivisceral transplantation. However, the limited number of patients and heterogeneous outcome reporting precludes firm conclusions regarding long‐term functional durability and predictors of rejection.

## 1 Introduction

Abdominal wall transplantation (AWT) is a form of vascularized composite allotransplantation (VCA) involving transplantation of the full‐thickness abdominal wall, including skin, subcutaneous tissue, fascia, musculature, and vascular pedicles from a deceased donor to reconstruct otherwise irreparable abdominal wall defects.

Closing the abdominal wall can be challenging in patients with poor‐quality abdominal walls, a history of multiple operations, numerous enterocutaneous fistulae, trauma, congenital abdominal wall anomalies, or a size discrepancy between the donor and recipient after transplantation of large edematous organs (Alexandrides et al. 2000). In certain cases, such as intestinal transplantation, primary fascial closure has a success rate of approximately 50%–85%, while 20%–33% experience secondary wound dehiscence (Cipriani et al. 2007). Conventional techniques such as component separation, tissue expansion, regional flaps, and free tissue transfer may be insufficient to address larger abdominal wall defects and wound dehiscence complications (Alexandrides et al. 2000).

Abdominal wall transplantation (AWT) was first introduced by Levi et al. in 2003. It has since provided new solutions to complex and selected surgical issues and has helped prevent postoperative morbidity and mortality (Levi et al. 2003). This surgical technique involves the transfer of various abdominal tissues, including the peritoneum, rectus sheath, rectus abdominis muscles, and obliques, as well as fat and skin layers. Abdominal wall transplantation represents an established application of vascularized composite allotransplantation primarily performed in conjunction with intestinal transplantation. The procedure aims to restore both the functional rehabilitation and aesthetic restoration of the abdominal region. This requires meticulous surgical skills, immunosuppressive management, and multidisciplinary collaboration among transplant surgeons, plastic surgeons, and other healthcare professionals (Park and Eun 2018). However, this technique has several drawbacks including limited well‐matched donors, cost and resource intensiveness, surgical complexity, function of the transplanted abdominal wall, risk of graft rejection, vascular complications, as well as the long‐term outcomes and the durability of abdominal wall transplants (Honeyman et al. 2020).

Despite more than two decades of clinical experience, the evidence remains limited to case reports and small case series. Important questions regarding patient selection, operative techniques, immunosuppressive strategies, rejection patterns, functional outcomes, and long‐term graft durability therefore remain unresolved (Erdmann and Atia 2023; Reed et al. 2022; Bustos et al. 2022).

Therefore, we performed a systematic review with an individual patient data analysis to comprehensively summarize the published clinical experience with abdominal wall vascularized composite allotransplantation. Our objectives were to characterize patient demographics, indications, operative techniques, immunosuppressive strategies, postoperative outcomes, and rejection patterns, and to explore potential factors associated with graft rejection.

## 2 Methods

### 2.1 Study Design

This systematic review with an individual patient data analysis evaluated the concept of AWT in transplantation surgery. The study followed the Preferred Reporting Items for Systematic Reviews and Meta‐Analyses (PRISMA) statement (Page et al. 2021) and the recommendations from the Study Center of the German Society of Surgery (Kalkum et al. 2021). The study protocol has been registered with the International Prospective Register of Systematic Reviews (PROSPERO) under the registration number CRD42024520835.

### 2.2 Literature Search

PubMed, Web of Science, Scopus, and Embase were searched from database inception through December 2025. Reference lists of eligible articles and relevant reviews were also screened. No publication‐date or language restrictions were applied. The following PubMed search terms were used: (“Abdominal wall transplantation”[Title/Abstract] OR “Abdominal wall allotransplantation”[Title/Abstract] OR “abdominal wall graft”[Title/Abstract] OR “Abdominal wall‐vascularized composite allotransplantation”[Title/Abstract] OR “AW‐VCA”[Title/Abstract]). The complete database‐specific search strategies are provided in the Supporting Information.

### 2.3 Eligibility Criteria

The eligibility criteria were formulated using the PICO framework (Amir‐Behghadami and Janati 2020). The population comprised patients who underwent full‐thickness AWT, and the intervention was AWT without a comparator. Outcomes of interest included demographics, indications, and operative and postoperative outcomes. Studies reporting operative or postoperative outcomes of AWT were eligible without restrictions based on patient age, race, sex, publication date, or language. Studies involving non‐human populations, conference papers, systematic reviews or meta‐analyses, cadaveric studies, and reports that did not include all layers of the abdominal wall were excluded.

### 2.4 Study Selection

Two reviewers (M.S. and M.S.) independently screened titles and abstracts. After duplicate and irrelevant records were excluded, potentially eligible articles underwent full‐text assessment. Disagreements were resolved by discussion, and data were extracted from studies that met the eligibility criteria.

### 2.5 Data Extraction

Two reviewers (N.S. and M.S.) extracted the following data into a standardized spreadsheet: first author, publication year, study country, patient demographics, indication for AWT, immunosuppressive regimen, rejection episodes and treatment, operative characteristics, and postoperative complications.

### 2.6 Risk of Bias Assessment

The studies included in this analysis underwent a risk of bias (ROB) assessment based on a modification of a three‐criteria assessment reported by Murad et al. (Murad et al. 2018). The assessment was divided into four domains: selection, ascertainment, causality, and reporting. The risk of bias was evaluated as low, some concerns, or serious. An overall risk of bias was also determined. The overall risk of bias was considered “serious” if at least one domain was rated as “serious risk of bias” or if there were “some concerns” in three or more domains. The overall risk of bias was “some concerns” if there were “some concerns” in at least one domain. The overall risk of bias was “low” if all domains were rated as “low risk of bias”.

### 2.7 Statistical Analysis

Statistical analysis was performed using SPSS version 26 (IBM Corp., Armonk, NY, USA). Patient‐level data were extracted from the eligible reports and harmonized before analysis. Continuous variables were summarized as median and interquartile range (IQR), and categorical variables as numbers and percentages.

Associations between categorical variables and biopsy‐proven AWT rejection were examined using Pearson's *χ* ^2^ test. All tests were two‐sided, and a *p*‐value < 0.05 was considered statistically significant. Given the small sample size and exploratory nature of the analysis, the findings were interpreted cautiously.

## 3 Results

### 3.1 Study Characteristics

A total of 271 records were retrieved from the specified databases through December 2025. After removing duplicates (*n* = 161), 110 articles were screened. Of these, 69 records had irrelevant titles or abstracts, leaving 41 articles for full‐text screening. Of these, 12 articles were conference papers (Zanfi et al. 2012, 2019; Allin, Ceresa, Casey, et al. 2013; Schimmelpfennig and Kritzmire 2013; Vrakas, Casey, et al. 2013; Vrakas, Giele, et al. 2013; Vrakas et al. 2014, 2016, 2017, 2019; Allan et al. 2016; Fernández et al. 2022), and nine articles were not associated with AWT or its outcomes (Hollenbeck et al. 2011; Singh et al. 2014; Broyles et al. 2015; Gerlach, Vrakas, Holdaway, et al. 2016; Reddy et al. 2017; Justo et al. 2022; Navarro Fajardo et al. 2023; Zanfi et al. 2015; Paessler et al. 2023). Twenty articles were included in the final analysis (Cipriani et al. 2007; Levi et al. 2003; Bejarano et al. 2004; Selvaggi et al. 2004, 2009; Lauro et al. 2006, 2013; Zanfi et al. 2008; Allin, Ceresa, Issa, et al. 2013; Mannu and Vaidya 2013, 2014; Giele et al. 2014; Gerlach, Vrakas, Sawitzki, et al. 2016; Haveman et al. 2016; Olson et al. 2017; Weissenbacher et al. 2018; Erdmann et al. 2019; Atia, Hollins, Erdmann, et al. 2020; Trentadue et al. 2020; Reddy et al. 2023). The PRISMA flowchart of the study is presented in Figure 1. The included studies were published between 2003 and 2023. Eight studies were case reports and the remaining 12 were case series. The studies were carried out in the USA, Italy, the UK, and the Netherlands. Seventy‐five percent of the studies were conducted in three centers: Miami, Florida (Levi et al. 2003; Bejarano et al. 2004; Selvaggi et al. 2004); Bologna, Italy (Cipriani et al. 2007; Lauro et al. 2006, 2013; Zanfi et al. 2008); and Oxford, England (Allin, Ceresa, Issa, et al. 2013; Mannu and Vaidya 2013, 2014; Giele et al. 2014; Gerlach, Vrakas, Sawitzki, et al. 2016; Weissenbacher et al. 2018; Reddy et al. 2023). One study was multicenter between Italy and the USA (Selvaggi et al. 2009). Other studies were conducted in Groningen, the Netherlands (Haveman et al. 2016; Trentadue et al. 2020) or other centers in the USA (Olson et al. 2017; Erdmann et al. 2019; Atia, Hollins, Erdmann, et al. 2020). In the assessment of ROB, 50% of articles were found to have a low risk of bias, while the remaining articles exhibited some concerns. The results of the ROB assessment for each study are presented in the Supporting Information.

**FIGURE 1:**
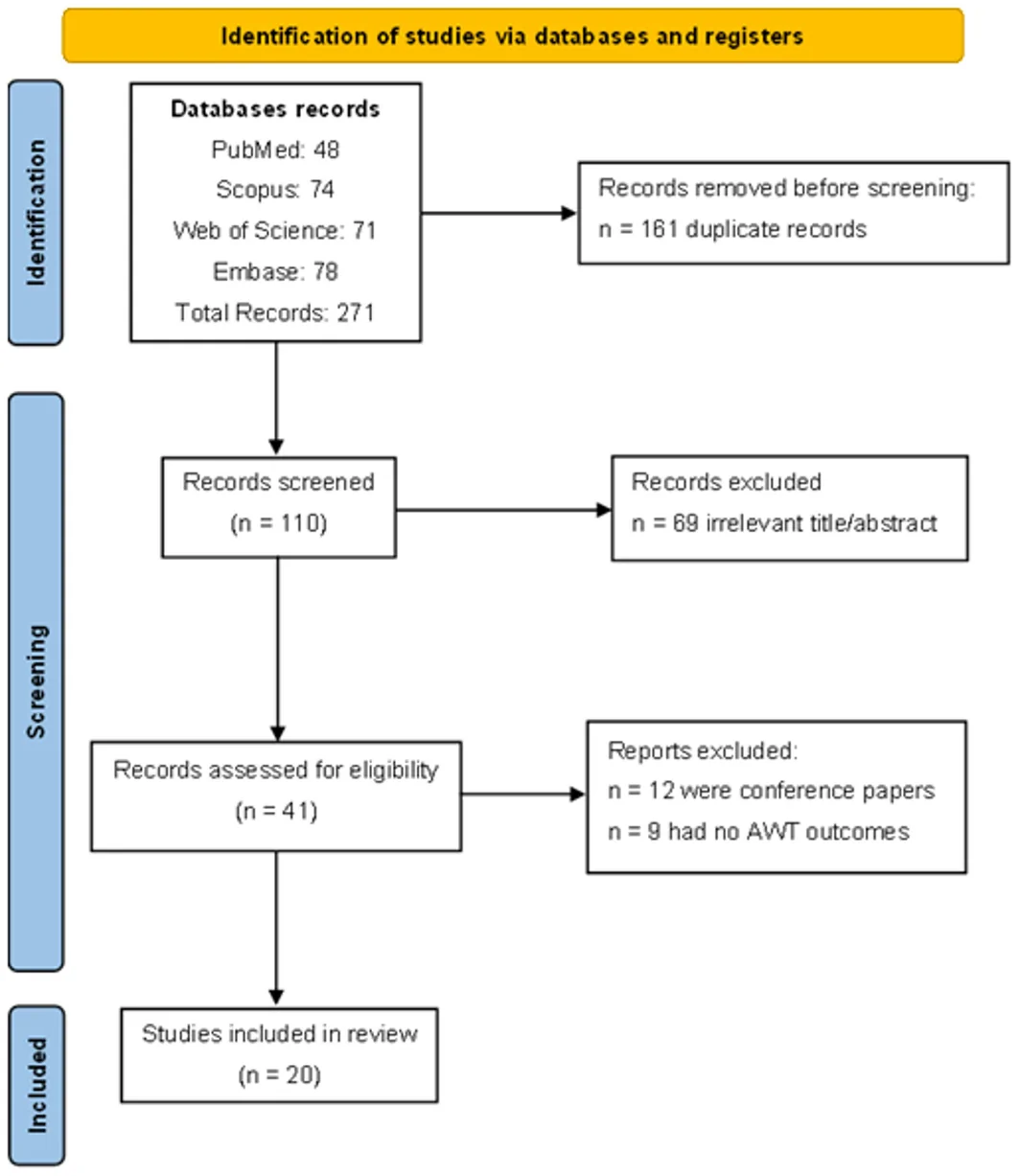
PRISMA flow diagram of study identification, screening, eligibility assessment, and inclusion.

### 3.2 Individual Patient Data Analysis

The analysis included 35 individual patients who underwent 37 full‐thickness AWT procedures. Two patients underwent repeat AWT. The combined Miami/Bologna experience reported 15 procedures in 14 patients; the aggregate report did not provide sufficient patient‐level information to determine the timing and indication for the repeated AWT, although two abdominal wall grafts in this series were removed because of vascular thrombosis (Selvaggi et al. 2009). The Oxford series reported one male patient who underwent retransplantation of both the intestinal and abdominal wall grafts 30 months after the first procedure because of ileus caused by encapsulating intestinal fibrosis (Gerlach, Vrakas, Sawitzki, et al. 2016). Sample sizes ranged from 1 to 18 patients. The median age was 37.5 years (IQR, 17.9), 20 patients (57.1%) were male, and 29 (82.9%) were adults. Follow‐up ranged from 1 to 7 years. General study characteristics are presented in Table 1.

**TABLE 1:** General characteristics of included studies.

Included reports (references)	Year	Case report/series	Patients	Male/Female	Age > 18	Age < 18	Mean age (range)	Follow‐up (yr)	Number of AWT
Levi et al. 2003; Bejarano et al. 2004; Selvaggi et al. 2004, 2009	2003–2009	0/4	10	6/4	6	4	19.6 (1–53)	7	11
Cipriani et al. 2007; Lauro et al. 2006, 2013; Zanfi et al. 2008	2006–2013	1/3	4	2/2	3	1	32.2 (17–41)	2	4
Allin, Ceresa, Issa, et al. 2013; Mannu and Vaidya 2013, 2014; Giele et al. 2014; Gerlach, Vrakas, Sawitzki, et al. 2016; Weissenbacher et al. 2018; Reddy et al. 2023	2013–2023	2/5	18	11/7	18	0	37.5 (23–73)	2	19
Haveman et al. 2016; Trentadue et al. 2020	2016–2020	2/0	1	0/1	0	1	24	1	1
Olson et al. 2017; Erdmann et al. 2019; Atia, Hollins, Erdmann, et al. 2020	2017–2020	3/0	2	1/1	2	0	28 (19–37)	1	2

Abbreviation: AWT, abdominal wall transplantation.

### 3.3 Preoperative Characteristics

Indications for AWT were categorized as congenital defects (e.g., gastroschisis), vascular disorders (e.g., intestinal ischemia or vasculitis), large abdominal wall defects (including trauma, previous abdominal operations, and Crohn disease), malignancy (including Gardner syndrome, desmoid tumors, and pseudomyxoma peritonei), and motility disorders (including Hirschsprung disease and chronic intestinal pseudo‐obstruction). Large abdominal wall defects were the most frequent indication (17/35, 48.6%), followed by malignancy (11/35, 31.4%), motility disorders (3/35, 8.6%), vascular disorders (2/35, 5.7%), and congenital defects (2/35, 5.7%). All AWT procedures were performed in conjunction with intestinal transplantation: 23 patients (65.7%) received an isolated intestinal transplant and 12 (34.3%) received a multivisceral transplant. Alemtuzumab was used for induction in 32 patients (91.4%) and thymoglobulin in three (8.6%). Tacrolimus was used for maintenance in all patients, with mycophenolate added in three cases. These findings are summarized in Table 2.

**TABLE 2:** Perioperative characteristics of included studies.

Included reports (references)	Patients	Indications					Transplantation		Immunosuppression		Perfusion	
		Congenital defects	Vascular disorder	Large abdominal defects	Malignancies	Motility disorders	Multivisceral	Isolated bowel	Induction	Maintenance	Early distant perfusion	Anastomoses
Levi et al. 2003; Bejarano et al. 2004; Selvaggi et al. 2004, 2009	10	2	0	4	3	1	5	5	Alemtuzumab	Tacrolimus	No	Macrovascular
Cipriani et al. 2007; Lauro et al. 2006, 2013; Zanfi et al. 2008	4	0	1	0	1	2	0	4	Alemtuzumab	Tacrolimus	No	Microvascular
Allin, Ceresa, Issa, et al. 2013; Mannu and Vaidya 2013, 2014; Giele et al. 2014; Gerlach, Vrakas, Sawitzki, et al. 2016; Weissenbacher et al. 2018; Reddy et al. 2023	18	0	1	10	7	0	6	12	Alemtuzumab	Tacrolimus	Yes	Microvascular
Haveman et al. 2016; Trentadue et al. 2020	1	0	0	1	0	0	0	1	Thymoglobulin	Tacrolimus + Mycophenolate	Yes	Microvascular
Olson et al. 2017; Erdmann et al. 2019; Atia, Hollins, Erdmann, et al. 2020	2	0	0	2	0	0	1	1	Thymoglobulin	Tacrolimus + Mycophenolate	No	Macrovascular

### 3.4 Intraoperative Characteristics

The operative time was reported for 11 patients by two study groups in Miami and Bologna, indicating that it added almost 2 h to the surgical procedure, ranging from 105 to 150 min. The anastomosis of the abdominal wall was conducted through epigastric or iliac circumflex complex. In 12 patients (34.3%), anastomosis was performed, connecting the donor's deep inferior epigastric vessels to the recipient's iliac circumflex vessels. In the remaining 23 patients (65.7%), anastomosis was utilized, linking the donor's deep inferior epigastric vessels to the recipient's corresponding vessels using an arteriovenous (AV) loop technique. In two study groups of Oxford and Groningen, the forearm vessels were used as an early distant perfusion (*n* = 19). In this approach, the abdominal wall graft is temporarily revascularized by connecting its blood vessels to the recipient's forearm vessels. This interim perfusion maintains the viability of the graft while the primary organ transplantation proceeds, effectively minimizing the duration the graft remains without blood supply. Once the main transplantation is complete, the abdominal wall graft is repositioned and permanently revascularized in its intended location. This method has been reported to reduce ischemia–reperfusion injury and improve overall outcomes in AWT procedures (Fortunato et al. 2022). The exact method of incision was not specified; however, based on the provided figures, it appears that the abdominal wall incision was made in a circular or oval shape. Unfortunately, it was not discussed in any study on muscle sutures, fascia sutures, possible anchoring, and other surgical details. The results for intraoperative characteristics are presented in Table 2.

### 3.5 Postoperative Assessment

Thirteen patients (37.2%) developed biopsy‐proven AWT rejection. Eleven of these 13 patients (84.6%) received intravenous corticosteroids as first‐line treatment. One corticosteroid‐resistant episode was subsequently treated with alemtuzumab, thymoglobulin was added to corticosteroids in one patient, and vedolizumab was used in another. Graft‐versus‐host disease (GVHD) affecting the intestinal graft but not the abdominal wall occurred in one Bologna patient (Lauro et al. 2013) and one Oxford patient (Mannu and Vaidya 2014); in both cases, the intestine and abdominal wall came from the same donor. Olson et al. reported scleromatous changes compatible with chronic rejection or GVHD confined to the abdominal wall in a recipient whose intestinal and abdominal wall grafts came from different donors (Olson et al. 2017). No mortality directly attributable to AWT was reported. Postoperative complications are summarized in Table 3. None of the included reports described standardized rehabilitation, physiotherapy, abdominal binder or compression‐garment protocols, or objective functional assessment of the transplanted musculature, such as electromyography.

**TABLE 3:** Postoperative complications of abdominal wall transplantation (AWT) in included studies.

Included reports (references)	Patients	Rejection	Rejection treatment	Other complications
Levi et al. 2003; Bejarano et al. 2004; Selvaggi et al. 2004, 2009	10	4	IV Corticosteroids	
Cipriani et al. 2007; Lauro et al. 2006, 2013; Zanfi et al. 2008	4	—	—	GVHD of the intestine, without involvement of abdominal wall (Alexandrides et al. 2000)
Allin, Ceresa, Issa, et al. 2013; Mannu and Vaidya 2013, 2014; Giele et al. 2014; Gerlach, Vrakas, Sawitzki, et al. 2016; Weissenbacher et al. 2018; Reddy et al. 2023	18	7	IV Corticosteroids/Alemtuzumab	GVHD of the intestine, without involvement of abdominal wall (Alexandrides et al. 2000)
Haveman et al. 2016; Trentadue et al. 2020	1	1	Vedolizumab	
Olson et al. 2017; Erdmann et al. 2019; Atia, Hollins, Erdmann, et al. 2020	2	1	IV Corticosteroids + Thymoglobulin	GVHD or chronic rejection of abdominal wall (Alexandrides et al. 2000)

Abbreviations: GVHD, graft‐versus‐host disease; IV, intravenous.

Mortality reporting was heterogeneous across the included studies, and standardized mortality outcomes were not consistently available. Therefore, a reliable pooled mortality rate could not be calculated. No death was reported as directly attributable to the abdominal wall graft or to AWT‐specific complications. Reported deaths occurred mainly in the context of the underlying intestinal or multivisceral transplantation and associated complications.

Potential factors associated with biopsy‐proven AWT rejection are presented in Table 4. Rejection occurred in 7 of 22 patients aged > 30 years and 6 of 13 patients aged < 30 years (*p* = 0.396); in 11 of 32 patients receiving alemtuzumab and 2 of 3 receiving thymoglobulin (*p* = 0.268); in 5 of 12 patients with a macrovascular anastomosis and 8 of 23 with a microvascular anastomosis (*p* = 0.689); and in 8 of 19 patients with early distant perfusion and 5 of 16 without early distant perfusion (*p* = 0.508). None of the assessed factors was significantly associated with rejection. These exploratory findings should be interpreted cautiously because of the small sample size. The principal findings are summarized in Figure 2.

**TABLE 4:** Possible risk factors for rejection after AWT.

Variable	Rejection of AWT, *n*/*N* (%)	No rejection of AWT, *n*/*N* (%)	*p*
Age > 30	7/22 (31.8)	15/22 (68.2)	0.396
Age < 30	6/13 (46.2)	7/13 (53.8)	
Immunosuppression induction with Alemtuzumab	11/32 (34.4)	21/32 (65.6)	0.268
Immunosuppression induction with Thymoglobulin	2/3 (66.7)	1/3 (33.3)	
Macrovascular anastomosis technique	5/12 (41.7)	7/12 (58.3)	0.689
Microvascular anastomosis technique	8/23 (34.8)	15/23 (65.2)	
Having distant early perfusion	8/19 (42.1)	11/19 (57.9)	0.508
Not having distant early perfusion	5/16 (31.3)	11/16 (68.8)	

*Note:* Values are *n*/*N* (%). *p* values were calculated using Pearson's *χ* ^2^ test.

Abbreviation: AWT, abdominal wall transplantation.

**FIGURE 2:**
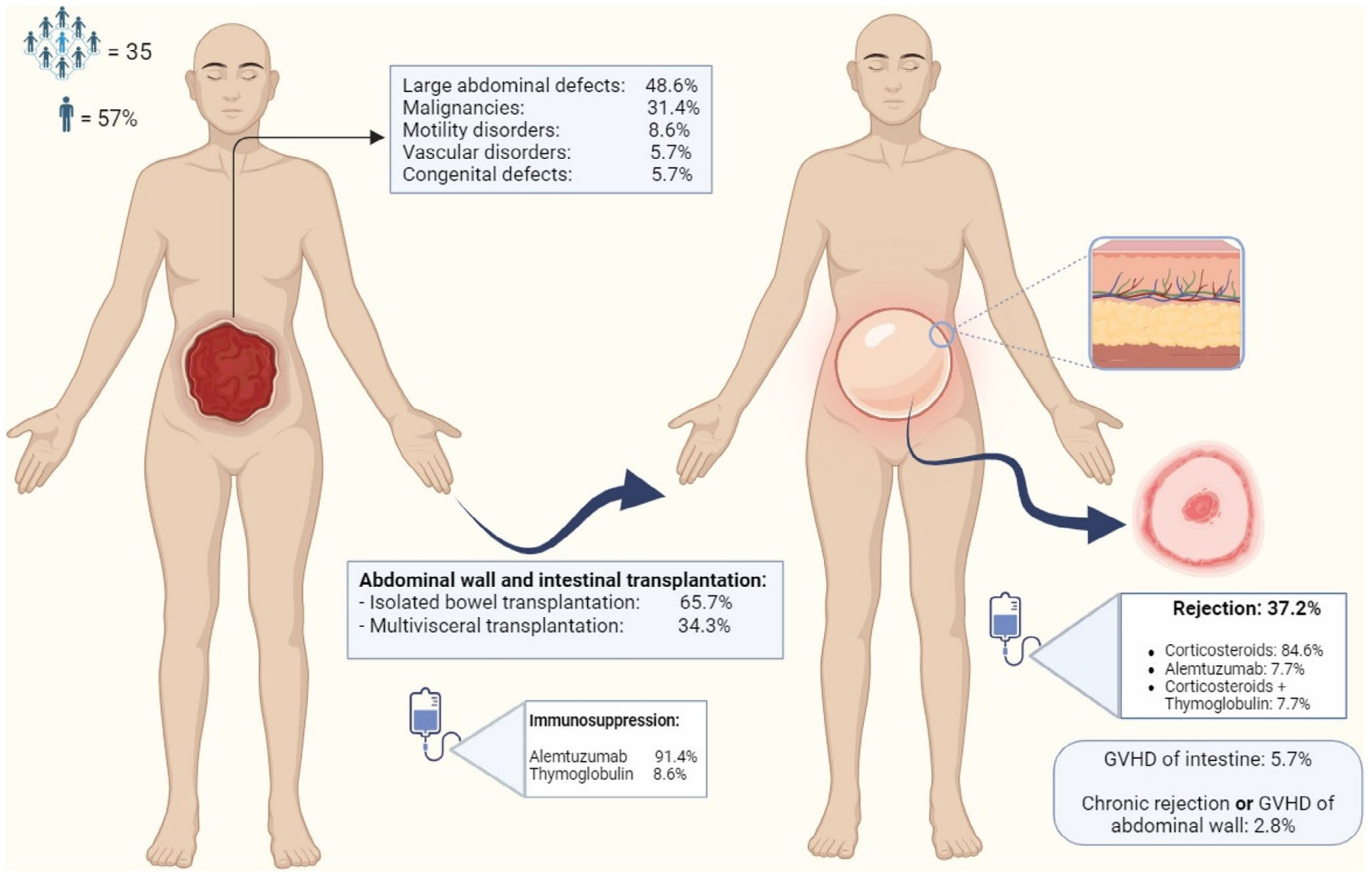
Summary of demographics, indications, transplantation types, immunosuppression, rejection treatment, and complications among 35 patients undergoing 37 AWT procedures. Treatment percentages are calculated among the 13 patients with biopsy‐proven rejection and are not mutually exclusive because treatment escalation occurred in some patients.

Although 23 patients underwent AWT with isolated intestinal transplantation and 12 underwent AWT with multivisceral transplantation, the included reports did not consistently provide patient‐level cross‐tabulated data linking visceral transplant type with biopsy‐proven AWT rejection. Therefore, a reliable comparison of AWT rejection rates between isolated intestinal and multivisceral transplantation could not be performed.

## 4 Discussion

The present systematic review of 20 studies involving 35 patients revealed that AWT was primarily indicated for large abdominal wall defects and malignancies, with most patients receiving alemtuzumab induction and tacrolimus maintenance. A biopsy‐proven AWT rejection rate of 37.2% was observed, mostly managed with intravenous corticosteroids, with no significant risk factors identified in the exploratory analyses. Overall, the review supports AWT as a feasible reconstructive option in selected patients undergoing intestinal or multivisceral transplantation, while highlighting the need for standardized long‐term outcome reporting.

A recent review of AWT outcomes following intestinal and multivisceral transplants reported findings that closely align with those of our current study (Reed et al. 2022). Nonetheless, our inclusion criteria include solely published journal articles, deliberately excluding unpublished conference publications to enhance the accuracy of the results. Typically performed in conjunction with visceral organ allotransplantation, these procedures have demonstrated effectiveness in aiding abdominal wall closure and acting as an indicator for rejection in associated visceral organ transplants. Conducting simultaneous abdominal wall and visceral organ transplantation extends the overall duration by approximately two hours (Molitor et al. 2018). If necessary, AWT could be delayed and carried out using tissue from a secondary donor (Molitor et al. 2018).

A key concern when adding an AWT component to intestinal or multivisceral transplantation is whether the skin‐containing vascularized composite allograft increases the immunologic risk to the visceral graft. Available comparative data do not suggest a clear increase in visceral graft rejection. Published intestinal transplant rejection rates are commonly reported at approximately 30%–50%, whereas previous AWT series reported intestinal rejection rates within or below this range. In the Oxford experience, intestinal rejection occurred in 17% of patients receiving combined AWT and intestinal transplantation compared with 36% of patients undergoing intestinal transplantation without AWT. Similarly, available data do not indicate increased donor‐specific antibody formation or sensitization after the addition of AWT. Therefore, although the evidence remains limited by small numbers and observational reporting, current data suggest that AWT can be added to intestinal or multivisceral transplantation without a clear increase in visceral graft rejection or patient risk.

The immunosuppressive approach for AWT generally followed the protocol used for the associated intestinal or multivisceral transplant. Alemtuzumab was used for induction in 32 patients (91.4%) and thymoglobulin in three (8.6%). Tacrolimus was used for maintenance in all patients, with mycophenolate added in three cases. Intravenous corticosteroid boluses remained the principal first‐line treatment for acute AWT rejection; refractory episodes were managed by intensifying immunosuppression with agents such as alemtuzumab, thymoglobulin, or vedolizumab, and extracorporeal photopheresis was used for persistent GVHD (Levi et al. 2003; Selvaggi et al. 2004; Allin, Ceresa, Issa, et al. 2013; Lauro et al. 2013; Gerlach, Vrakas, Sawitzki, et al. 2016; Trentadue et al. 2020).

Most included centers adopted intestinal transplantation immunosuppressive protocols, which frequently employ lymphocyte‐depleting induction therapy such as alemtuzumab or thymoglobulin followed by tacrolimus‐based maintenance while minimizing corticosteroid exposure. Steroid‐sparing protocols are commonly used to reduce long‐term corticosteroid‐related adverse effects while maintaining adequate immunosuppression in these highly complex transplant recipients (Levi et al. 2003; Selvaggi et al. 2004; Allin, Ceresa, Issa, et al. 2013; Lauro et al. 2013; Gerlach, Vrakas, Sawitzki, et al. 2016).

The retrieval of the abdominal wall poses no risk to the harvesting of other organs, as they possess distinct vascular supplies. However, perfusion of the transplanted abdominal wall is of great importance. Microsurgical techniques can be employed for the transfer of the abdominal wall, utilizing the iliac, femoral, or deep inferior epigastric vessels. Results of a systematic review revealed that AWT presents a technically feasible option for challenging abdominal wall reconstruction scenarios (Atia, Hollins, Shammas, et al. 2020). This technique is primarily utilized to reconstruct large or complex abdominal wall defects that cannot be effectively repaired using conventional methods. AWT not only restores the structural integrity of the abdominal wall but also aims to improve its functionality, especially when nerve integration is achieved. This approach has been particularly beneficial for patients undergoing intestinal or multivisceral transplantation, where traditional reconstructive options may be limited (Bustos et al. 2022). The literature review identified four revascularization techniques with varying outcomes, highlighting the need for ongoing refinement and optimization as the field of AWT continues to advance. Accordingly, Levi et al. published a pioneering study outlining the technical feasibility of AWT by connecting the donor's external iliac and inferior epigastric vessels to the recipient's common iliac vessels in an end‐to‐side anastomosis technique (Levi et al. 2003). Four years later, a microsurgical method involving an end‐to‐end anastomosis between donor and recipient inferior epigastric vessels was introduced by Cipriani (Cipriani et al. 2007). Banking the abdominal wall allograft in the forearm was first described by Giele et al. as a unique approach to reduce graft ischemia time (Giele et al. 2014). More recently, Erdmann et al. described a novel strategy utilizing an AV loop for simultaneous revascularization of abdominal wall and visceral transplants, aiming to reduce ischemia and operative time, thereby eliminating the need for additional procedures (Erdmann et al. 2019). However, our results revealed no significant rejection difference either in patients with lower ischemia time, through distant perfusion, or in patients with macro or microvascular techniques.

It has been suggested that skin could be representative of forthcoming rejection in other transplanted viscera as the earliest or even solitary signs of rejection that could be stopped with prompt preventive strategies to rescue other transplanted structures (Cipriani et al. 2007; Levi et al. 2003; Petruzzo and Dubernard 2011; Zamfirescu et al. 2009; Morelon et al. 2012). Frequent allograft rejection episodes to the skin component of composite tissue allotransplant (CTA) may be attributed to increased antigenic skin load in these transplants because powerful immunologic responses may be seen compared with other components. Previous studies have revealed that cutaneous evidence of rejection could happen independent of intestinal involvement in cases with VCA receiving isolated bowel or multi‐visceral transplants (Cipriani et al. 2007; Levi et al. 2003; Allin, Ceresa, Issa, et al. 2013). The merging of two grafts with high immunogenicity, namely the intestine and VCA may pose an unfamiliar immunological threat for allograft rejection and the development of GVHD (Selvaggi et al. 2009). In these cases, determining the greater immunological challenge posed by each graft is challenging. The immunogenicity of bowel mucosa is comparable to that of skin, and the disparity in the onset of rejection between skin and bowel may be negligible (Zhang et al. 1996). In the Miami studies, isolated rejection episodes were observed twice for bowel and twice for abdominal wall transplants, highlighting the difficulty in discerning a significant difference (Levi et al. 2003; Selvaggi et al. 2009). Two patients from Bologna experienced bowel rejection, while no rejection of the abdominal wall was observed (Cipriani et al. 2007). Researchers from Oxford documented four cases of isolated abdominal wall rejection and only one occurrence of simultaneous rejection involving both the abdominal wall and the bowel (Giele et al. 2014, 2016; Gerlach, Vrakas, Sawitzki, et al. 2016). Recent observations indicate that abdominal wall and intestinal allotransplantation can exhibit independent rejection patterns with similar frequencies and frequently as isolated incidents. We also assessed possible causes of rejection in patients but through assessing age, immunosuppression regimens, surgical technique or perfusion type no risk factors were found for rejection. Further studies are needed to assess the possible risk factors for AWT rejection.

To our knowledge, this study represents the first systematic review with an individual patient data analysis dedicated exclusively to abdominal wall vascularized composite allotransplantation. By extracting and analyzing patient‐level data from all eligible published reports, we were able to characterize indications, operative strategies, immunosuppressive regimens, rejection patterns, and postoperative outcomes in greater detail than previously possible. Furthermore, this is the first study to explore potential predictors of graft rejection using pooled individual patient data. These findings provide clinicians with a comprehensive overview of the current evidence and identify important priorities for future research. Despite the strengths of our study, several limitations warrant acknowledgment. Firstly, the relatively small sample size of 35 patients may limit the generalizability of our findings and hinder the detection of rare adverse events or subtle risk factors. Furthermore, the absence of long‐term follow‐up data in some cases may obscure the full spectrum of outcomes, including late complications or graft survival rates beyond the immediate postoperative period. Additionally, our reliance solely on published literature may introduce publication bias and overlook relevant data from ongoing or unpublished studies. Moreover, the heterogeneity among the included studies in terms of surgical techniques, immunosuppressive regimens, and patient characteristics may introduce confounding variables that could not be fully accounted for in our analysis. In addition, several relevant aspects were not investigated, including the size and strength of the AWT, its stability, and the potential for postoperative hernia formation or other surgical complications. Likewise, psychological perceptions of the new abdominal wall, psychosocial factors, and aesthetic considerations were not addressed.

The main contribution of the present study is not the identification of a new therapeutic principle, but an independent synthesis of the published peer‐reviewed experience with patient‐level data extraction. This approach allowed standardized reassessment of indications, operative strategies, immunosuppression, rejection episodes, and exploratory rejection‐associated factors across the available literature. Given that the field remains limited to small case series and reports from highly specialized centers, independent analyses may help validate previously reported observations and identify areas where standardized prospective reporting is still needed.

The lack of consistent patient‐level cross‐tabulation of visceral transplant type and AWT rejection further prevented a reliable subgroup comparison.

## 5 Conclusions

AWT is currently reserved for carefully selected patients with extensive, otherwise irreparable abdominal wall defects, most often in conjunction with intestinal or multivisceral transplantation. The available evidence supports its technical feasibility and ability to achieve abdominal closure, but the small and heterogeneous evidence base prevents definitive conclusions regarding comparative effectiveness, long‐term durability, function, and predictors of rejection. Prospective multicenter data collection with standardized reporting of surgical, immunological, functional, and patient‐reported outcomes is needed to refine patient selection and optimize long‐term care.

## Author Contributions

M.S. conceived and coordinated the study, developed the methodology, conducted the literature search, participated in study screening and data extraction, performed the statistical analysis and visualization, and drafted the manuscript. N.S. contributed to the methodology, data extraction, data validation, analysis and interpretation, visualization, and drafting and revision of the manuscript. M.S. participated in study screening, data validation, and critical revision of the manuscript. E.K. and C.M. contributed to the interpretation of the findings and critically revised the manuscript. A.M., U.K., and C.K. provided expert input, supervised the study, interpreted the findings, and critically revised the manuscript. C.K. additionally contributed to the study conception and project administration. All authors read and approved the final manuscript and agree to be accountable for all aspects of the work.

## Funding

The authors have nothing to report.

## Ethics Statement

The authors have nothing to report.

## Conflicts of Interest

The authors declare no conflicts of interest.

## Supporting information


**Table S1:** ROB assessment of the included studies.

## Data Availability

The data underlying this systematic review were obtained from the published studies cited in the manuscript. The extracted dataset is available from the corresponding author upon reasonable request.
