# Supplementary material for: Insights Into Abdominal Wall Vascularized Composite Allotransplantation: An Individual Patient Data Analysis
Source: Microsurgery. 2026 Nov 14;46(8):e70346. doi: 10.1002/micr.70346 (PMC13768980; doi:10.1002/micr.70346)
Supplement: Supplementary file 1 [file MICR-46-e70346-s001.docx]

Insights into Abdominal Wall Vascularized Composite Allotransplantation: An Individual Patient Data Analysis

Mohammadsadegh Sabagh¹˒²˒³*, MD; Nastaran Sabetkish¹, MD; Mohammadamin Shahrbaf¹, MD;

Elias Khajeh¹, MD; Christoph Michalski¹, MD; Arianeb Mehrabi¹, MD; Ulrich Kneser²˒³, MD;

Christoph Koepple²˒³, MD

1. Department of General, Visceral and Transplantation Surgery, Heidelberg University Hospital, Heidelberg, Germany.

2. Department of Plastic, Hand and Reconstructive Surgery, BG Trauma Center Ludwigshafen, Ludwigshafen, Germany.

3. Department of Hand and Plastic Surgery, University of Heidelberg, Heidelberg, Germany.

**Corresponding author information:**

Mohammadsadegh Sabagh, MD; Department of Plastic, Hand and Reconstructive Surgery, BG Trauma Center Ludwigshafen, Ludwigshafen, Germany.

Email: Mohammadsadegh.Sabagh@bgu-ludwigshafen.de

Tel: +4962168108989

**Search strategy**

| "Abdominal wall transplantation"[Title/Abstract] OR "Abdominal wall allotransplantation"[Title/Abstract] OR "abdominal wall graft"[Title/Abstract] OR "Abdominal wall–vascularized composite allotransplantation"[Title/Abstract] OR "AW-VCA"[Title/Abstract] | PubMed |
| --- | --- |
| TITLE-ABS-KEY ("Abdominal wall transplantation" OR "Abdominal wall allotransplantation" OR "abdominal wall graft" OR "Abdominal wall–vascularized composite allotransplantation" OR "AW-VCA") | Scopus |
| “Abdominal wall transplantation” OR “Abdominal wall allotransplantation” OR “abdominal wall graft” OR “Abdominal wall–vascularized composite allotransplantation” OR “AW-VCA” (Topic) | WOS |
| 'abdominal wall transplantation':ti,ab,kw OR 'abdominal wall allotransplantation':ti,ab,kw OR 'abdominal wall graft':ti,ab,kw OR 'abdominal wall–vascularized composite allotransplantation':ti,ab,kw OR 'aw-vca':ti,ab,kw | Embase |

**Risk of bias assessment**

Table S1. ROB assessment of the included studies

| Study | Selection | Ascertainment | Causality | Reporting | Overall |
| --- | --- | --- | --- | --- | --- |
| Levi et al. 2003 (4) | Low | Low | Low | Low | Low |
| Bejarano et al. 2004 (33) | Low | Low | Some concerns | Low | Some concerns |
| Selvaggi et al. 2004 (34) | Low | Low | Some concerns | Low | Some concerns |
| Lauro et al. 2006 (35) | Low | Low | Some concerns | Low | Some concerns |
| Cipriani et al. 2007 (2) | Low | Low | Low | Low | Low |
| Zanfi et al. 2008 (36) | Low | Low | Some concerns | Some concerns | Some concerns |
| Selvaggi et al. 2009 (37) | Low | Low | Low | Some concerns | Some concerns |
| Allin et al. 2013 (38) | Low | Low | Low | Low | Low |
| Lauro et al. 2013 (39) | Low | Some concerns | Low | Low | Some concerns |
| Mannu et al. 2013 (40) | Low | Some concerns | Low | Low | Some concerns |
| Giele et al. 2014 (41) | Low | Low | Low | Low | Low |
| Mannu et al. 2014 (42) | Low | Some concerns | Low | Low | Some concerns |
| Gerlach et al. 2016 (43) | Low | Low | Low | Low | Low |
| Haveman et al. 2016 (44) | Low | Low | Low | Low | Low |
| Olson et al. 2017 (45) | Low | Some concerns | Low | Some concerns | Some concerns |
| Weissenbacher et al. 2018 (46) | Low | Some concerns | Low | Some concerns | Some concerns |
| Erdmann et al. 2019 (47) | Low | Low | Low | Low | Low |
| Atia et al. 2020 (48) | Low | Low | Low | Low | Low |
| Trentadue et al. 2020 (49) | Low | Low | Low | Low | Low |
| Reddy et al. 2023 (50) | Low | Low | Low | Low | Low |
